# A New Strain of *Christensenella minuta* as a Potential Biotherapy for Obesity and Associated Metabolic Diseases

**DOI:** 10.3390/cells10040823

**Published:** 2021-04-06

**Authors:** Wilfrid Mazier, Katy Le Corf, Ccori Martinez, Héloïse Tudela, Déborah Kissi, Camille Kropp, Chrislain Coubard, Marion Soto, Frédéric Elustondo, Georges Rawadi, Sandrine P. Claus

**Affiliations:** 1 Ysopia Bioscience, 17 place de la Bourse, 33076 Bordeaux, France; wilfrid.mazier@ysopia.bio (W.M.); katy.lecorf@ysopia.bio (K.L.C.); ccorimartinez@gmail.com (C.M.); heloise.tudela@ysopia.bio (H.T.); deborah.kissi@ysopia.bio (D.K.); camille.kropp@ysopia.bio (C.K.); chrislain.coubard@gmail.com (C.C.); marion.soto@ysopia.bio (M.S.); Frederic.elustondo@ysopia.bio (F.E.); georges.rawadi@ysopia.bio (G.R.); 2 Micalis Institute, INRAE, AgroParisTech, University Paris-Saclay, 78350 Jouy-en-Josas, France

**Keywords:** obesity, microbiome, metabolic disorders

## Abstract

Obesity is associated with gut microbiota dysbiosis, characterized by a high Firmicutes/Bacteroidetes ratio. Gut-dwelling bacteria of the Christensenellaceae family have been proposed to act as keystones of the human gut ecosystem and to prevent adipogenesis. The objectives of the present study were to demonstrate the antiobesity potential of a new strain of *Christensenella minuta* in preclinical models and explore related mechanisms of action. The antiobesity potential of *C. minuta* DSM33407 was assessed in a diet-induced obesity mouse model. Changes in hepatic lipid metabolism were explored using targeted transcriptomics. Effects on gut microbiota were further assessed in a humanized Simulator of the Human Intestinal Microbial Ecosystem (SHIME^®^) model inoculated with obese fecal samples. Shotgun metagenomics was applied to study microbial community structures in both models. *C. minuta* DSM33407 protected from diet-induced obesity and regulated associated metabolic markers such as glycemia and leptin. It also regulated hepatic lipid metabolism through a strong inhibition of de novo lipogenesis and maintained gut epithelial integrity. In the humanized SHIME^®^ model, these effects were associated with modulations of the intestinal microbiota characterized by a decreased Firmicutes/Bacteroidetes ratio. These data indicate that *C. minuta* DSM33407 is a convincing therapeutic candidate for the management of obesity and associated metabolic disorders.

## 1. Introduction

There is growing interest in the gut commensal gram-negative Clostridiales of the *Christensenellaceae* bacteria, as an increasing number of studies have recently reported their consistent association with leanness and health. *Christensenella minuta* was the first species described in this family in 2012 [1]. In 2014, Goodrich et al. identified *C. minuta* as the most heritable bacterial taxon in humans [2]. They also suggested the therapeutic anti-obesity potential of *C. minuta*, demonstrating a causal link between *C. minuta* and a reduction of visceral fat mass accumulation in a murine model.

In humans, *Christensenellaceae* have been repeatedly associated with leanness in many observational population studies [3,4,5,6]. In addition, it has been shown that *Christensenellaceae* bacteria considerably increase during weight loss, suggesting a close relationship between the presence of these bacteria in the gut ecosystem and the regulation of energy metabolism [7]. In the elderly, the *Christensenellaceae* have been associated with healthy aging [8,9]. Other conditions where *Christensenellaceae* have been identified as missing microbes include Crohn’s disease [10,11], ulcerative colitis [12], and irritable bowel syndrome [13], suggesting that they may also play a protective role in the regulation of inflammation.

In observational studies, beyond their association with leanness, *Christensenellaceae* have been reported as strongly anticorrelated with relevant clinical markers such as circulating LDL levels [5,14], hypertriglyceridemia [5,6], elevated blood pressure [15,16], and circulating alanine transferase (ALT) levels, a marker of hepatic function [6]. As a dysbiosis of the gut microbiome has been suggested as a causal factor of metabolic disorders associated with obesity [17], these observations may be indicative of a key role played by bacteria of the *Christensenellaceae* family in regulating energy balance and metabolic homeostasis. Hence, bacteria of the *Christensenellaceae* family hold great potential for therapeutic use as live biotherapeutic products to treat obesity and associated metabolic disorders.

In this article, we describe a novel specimen of *C. minuta* isolated from a healthy human donor and question its therapeutic potential in a diet-induced obesity (DIO) mouse model. We further evaluate the keystone function of this specific strain in an in vitro model of human intestine inoculated with microbiota derived from donors with obesity.

## 2. Materials and Methods

### 2.1. Bacteria Phenotypic Characterization

#### 2.1.1. Culture Growth

DSM 33407 was cultured on prereduced Gifu anaerobic modified medium (GAMm, Hyserve, Uffing, Germany) during three days at 37 °C in anaerobic atmosphere (H_2_ 5%, CO_2_ 5%, N_2_ 90%).

#### 2.1.2. Microbial Characterization

All phenotypic tests were performed in triplicate and used reference strains *C. minuta* DSM 22607 and *Bacteroides fragilis* DSM 2151 as controls. Biochemical characterization of DSM 33407 was performed using API 20A anaerobic test kit (Biomérieux, Marcy l’Etoile, France) following the manufacturer’s instructions. Briefly, strips were inoculated with 100 μL API 20 A medium and incubated for 48 h at 37 °C in anaerobic conditions prior to visual evaluation at ambient atmosphere.

Oxidase activity was determined with oxidase test strips (VWR, Fontenay-sous-Bois, France) by measuring the strip color shift after adding a drop of DSM 33407 liquid culture.

Catalase activity was determined by adding some drops of hydrogen peroxide 3% and 12% solution to a DSM 33407 colony.

Gram-staining and spore-forming assays were performed as previously described [18].

Bile acid and pH tolerance were evaluated using both GAM modified broth and 1.5% agar. Liquid broth contained 0%, 0.2%, 2%, 4%, 6%, 8% *w*/*v* Oxgall (Difco Laboratories supplied by BD Diagnostics, Le Pont de Claix, France) equivalent to 0%, 2%, 20%, 40%, 60%, and 80% bile, respectively. Agar plates were buffered at pH levels 4, 5, 6, 7, and 8. Broth and agar media were previously pre-reduced and inoculated using a bacteria suspension equivalent to a 0.5 McFarland standard (approximately 108 CFU/mL). In the case of agar plates, 3 μL of inoculum was spotted and incubated anaerobically at 37 °C for five days. Growth was assessed visually as (+) growth, (−) no growth, or (+/−) growth substantially inhibited relative to the growth control but not completely devoid of growth. In the case of liquid cultures, 10 μL aliquot of inoculum was used to inoculate GAM modified broth tubes and incubated anaerobically at 37 °C for four days. Growth was evaluated by measuring optical density at 600 nm (Biochrom WPA Biowave, Cambridge, UK).

Antibiotic resistance testing was performed using the minimum inhibitory concentration (MIC) using reference agar dilution method according to the Clinical and Laboratory Standards Institute (CLSI) [19,20]. The following antibiotic stock solutions were prepared and sterilized: Ampicillin (Merck, Darmstadt, Germany), Ceftriaxone (USP, Frederick, MD, USA), Chloramphenicol (Merck, Darmstadt, Germany), Clindamycin (Merck, Darmstadt, Germany), Meropenem (USP, Frederick, MD, USA), Metronidazole (Merck, Darmstadt, Germany), Moxifloxacin (ChemPacific, Baltimore, MD, USA), Piperacillin (Merck, Darmstadt, Germany), Tazobactam (USP, Frederick, MD, USA), Tetracycline (Merck, Darmstadt, Germany). Brucella agar (BD BBL, Le Pont de Claix, France) supplemented with 5 μg/mL hemin (Merck, Darmstadt, Germany), 1 μg/mL vitamin K1 (Merck, Darmstadt, Germany), and 5% (*v*/*v*) laked sheep blood (Hemostat, Dixon, CA, USA) was used as the test medium. Drug-supplemented agar plates at different dilutions were prepared and prereduced in the anaerobic glovebox prior to inoculation. An inoculum of DSM 33407 was suspended to the equivalent of a 0.5 McFarland standard in prereduced saline using a turbidity meter (Siemens Healthineers Dade Berhing MicroScan, Erlangen, Germany). Each bacterial cell suspension was then transferred to wells in a stainless-steel replicator block which was used to inoculate the test plates. The prongs on the replicator delivered approximately 1–2 μL of inoculum to the agar surface which corresponded to approximately 10^5^ CFU/spot. Plates were incubated at 37 °C for six days in anaerobic conditions. The MIC values were determined according to CLSI guidelines and were interpreted as susceptible (S), intermediate (I), or resistant (R) based on CLSI anaerobe breakpoints [19,20].

#### 2.1.3. 16S Genotyping and Phylogenetic Analysis

Total DNA was extracted from a *C. minuta* DSM33407 pellet equivalent to 10^9^ bacterial cells using MagAttract HMW DNA kit according to the manufacturer’s instructions (Qiagen, Courtaboeuf, France). DNA was quantified using a Nanodrop spectrophotometer (Thermo Fisher Scientific, Illkirch, France) and diluted to a final concentration of 20 ng μL^−1^ for 16S rRNA PCR amplification using universal primers (sequences in Supplementary Material) (hybridization temperature 55 °C) providing a total fragment length of 1500 pb. Amplified products were sequenced by Genewiz (Paris, France). The entire 16S sequence is publicly available on GenBank using the following access number: MW751847. The 16S rRNA sequence was blasted against refseq_RNA database (NCBI). A maximum likelihood tree was built using MEGA-X 10.1.8 [21]. Details are described in Supplementary Material.

### 2.2. Animal Assays

All animal experiments were performed in an accredited Contract Research Organization (CRO, Biomeostasis, La Penne sur Huveaune, France) in accordance with governmental ethical guidelines (accreditation number C13-055-31; APAFIS#16306/APAFIS#17846/APAFIS#23402). A total of 120 five-week-old C57Bl/6J male mice were included in four independent studies (30 animals per study) using the same diet-induced obesity (DIO) mouse model. Following a two-week acclimation period, all animals were randomly allocated to three treatment groups of 10 individuals each, based on body weight. In all four studies, 20 animals were switched to a high-fat, sucrose-rich diet (HFD group, 45%cal fat/20%cal sucrose, D12451i, Research Diet) on the day of treatment initiation. The other animals were maintained on control diet (NC group, n = 10, A04, SAFE). Half of the HFD group (n = 10) received the *Christensenella minuta* DSM33407 (liquid formulation, PBS 1×, 1% glycerol, 2.10^9^ CFU/day) while the other half received the vehicle only. The NC group received the vehicle only. For all experiments, food intake was measured every three days and caloric intake (kcal) was calculated from diets energy density (kcal/g). Treatment duration lasted from 4 to 12 weeks, depending on study endpoints. More details about housing, sample collection, and analyses can be found in Supplementary Material.

### 2.3. Triple SHIME^®^ Model

Obese fecal samples presenting very low (<10^6^ copies/g of stool) abundance of *Christensenella minuta* were used for this study and the Triple SHIME^®^ experiment was performed using standard conditions [22] (see supplemental for more details). Briefly, three Triple SHIME^®^ models were run in parallel to simulate the stomach, small intestine, and two colonic regions (proximal and distal colon). The system was fed daily for three consecutive weeks. The treatment period was followed by one week of product washout aiming at evaluating the persistence of treatment effects. A weekly sampling allowed assessing the presence of DSM33407 and monitoring variations of the ecosystem (see Supplemental Material).

### 2.4. Shotgun Metagenome Sequencing

#### 2.4.1. DNA Extraction Procedure

DNA was extracted from 0.2 mg of sample using DNeasy Power Soil Pro according to the manufacturer’s instructions and quantified using Qubit 4 fluorometer and Qubit™ dsDNA HS Assay Kit (Thermofisher Scientific, Illkirch, France).

#### 2.4.2. DNA Library Preparation

DNA libraries were prepared using the Nextera XT DNA Library Preparation Kit (Illumina, Evry, France and Nextera Index Kit (Illumina, Evry, France) following the manufacturer’s protocol with slight modifications that are described in Supplemental Material.

#### 2.4.3. Sequencing on Illumina HiSeq4000

DNA libraries were pooled together proportionally based on target read depth. Quality check of the pooled library was done with a Tapestation (Agilent Technologies, Les Ulis, France) to examine fragment size and concentration of the pool. Then, the pooled library was diluted and denatured using standard protocol. Finally, paired-end sequencing (2 × 150 bp) was performed on Illumina HiSeq4000. Samples received an average read depth of 4.99 million reads.

#### 2.4.4. Microbiota Analysis

Taxonomic classification of microbial NGS reads was performed using proprietary methods and catalog from CosmosID Inc (Rockville, MD, USA). The microbiota analysis was performed at the family level. The annotated and normalized dataset was exported to R (v.4.0.3) for multivariate analysis. To avoid bias induced by the large proportion of christensenella-related reads, the *Christensenellaceae* variable was removed from the dataset. The latter was normalized again, and clusters were identified using principal component analysis on mean centered and unit variance scaled data. A PERMANOVA on Bray–Curtis distances was calculated using the “adonis” function from the “vegan” package (v. 2.5-7). Pairwise PERMANOVA comparisons were calculated and the *p* value adjusted using Bonferroni’s correction.

### 2.5. Statistical Analysis

Univariate statistics were performed with Prisms 8 (Graphpad, San Diego, CA, USA). Normality distribution was assessed using Shapiro–Wilk or Kolmogorov–Smirnov test. Considering interindividual variability in body weight gain in response to HFD, an outlier identification (Grubb’s test) was systematically made on normalized body weight gain data. Animals with aberrant body weight changes were discarded from analysis. Data from body weight changes, food efficiency, and glycemia assessment were analyzed by two-way ANOVA repeated measures followed by Dunnett’s multiple comparisons or Fischer’s LSD test. Data from body composition and plasma markers were analyzed by one-way ANOVA followed by Holm–Sidak’s multiple comparisons. Data from histological scoring were analyzed using pair-wise Mann–Whitney tests. Regarding SHIME^®^ data, due to different numbers of samples depending on timepoints, repeated measures ANOVA could not be handled. Therefore, analyses were made fitting a mixed model of Restricted Maximum Likelihood (REML), followed by uncorrected Fischer’s LSD tests. For all analyses, the significance threshold was placed at *p* = 0.05. In the text and figures, data are represented as mean ± SEM.

## 3. Results

### 3.1. Strain Isolation and Characterization

**Figure 1:**
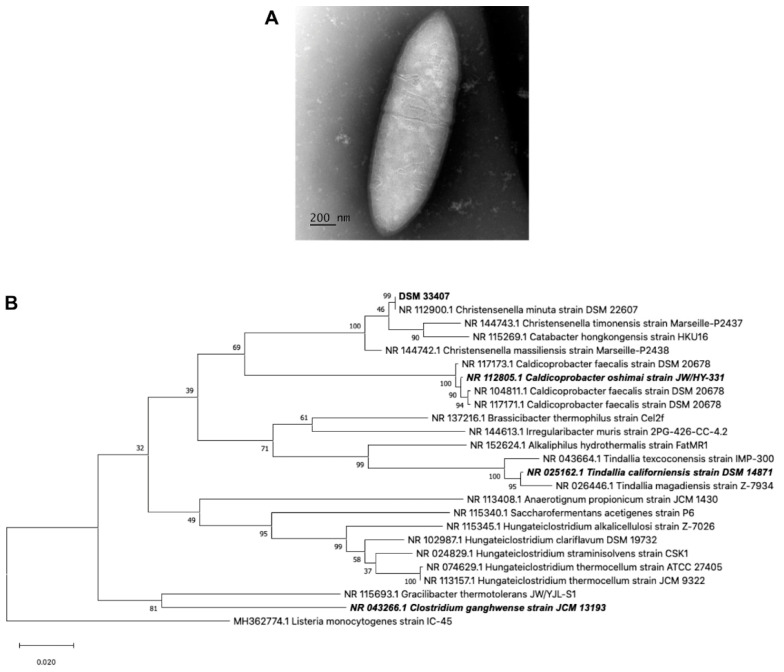
Classification of new strain DSM33407 as a strain of *Christensenella minuta*. (**A**) TransmisScheme 33407. (**B**) Maximum likelihood tree showing the closest relative of strain DSM33407 based on 16S rRNA gene sequence. *Listeria monocytogenes* strain IC-45 was used as outgroup. Numbers on the branches are bootstrap values validated using 1000 iterations. In bold is the DSM33407 16S rRNA sequence; in bold and italics are the 16S rRNA sequences identified as closely related to *C. minuta* DSM 22607 in the original type strain publication by Morotomi et al. based on 16S rRNA sequences [1].

Strain DSM33407 was isolated from a fecal sample of a young (<35 years old) healthy human donor using a culturomics approach as described by [23]. The 16S rRNA coding region was amplified (total fragment length of 1116 pb) and matched with 99% identity with *Christensenella minuta* strain DSM22607 (NR 112900.1). *Christensenella minuta* was first described by Morotomi et al. (2012), where the closest relatives were identified as *Caldicoprobacter oshimai*, *Tindallia californiensis*, and *Clostridium ganghwense* [1]. Thus, a phylogenetic tree was built using the 16S rDNA of these three species together with the 20 top hits identified by BLAST, and *Listeria monocytogenes* as outgroup (Figure 1B). This analysis confirmed that the new isolate clustered very closely to *Christensenella minuta* DSM22607.

Strain DSM33407 was strictly anaerobic, nonmotile, non-spore-forming, negative for oxidase and catalase activities (Table 1), and stained Gram-negative (Supplementary Figure S1). Colonies were circular, raised, opaque, and tiny (<1 mm). Cell morphology observed using TEM microscopy revealed short straight rods with tapered ends, as described for *Christensenella minuta* DSM22607 [1], occurring singly or in pairs (Figure 1A). Average dimensions were 1.77 ± 0.34 µm length and 0.52 ± 0.03 µm wide. Membrane thickness was 35 ± 5 nm, which is consistent with a Gram-negative species.

DSM33407 was negative for most enzyme tests except acidification of glucose, salicin, xylose, rhamnose, and arabinose (Table 1), which is consistent with the description of *Christensenella minuta* [1]. The susceptibility of DSM33407 to a variety of antibiotics was assessed in accordance with guidelines from the Clinical and Laboratory Standards Institute (Table 2) [19,20]. Strain DSM33407 was resistant to tetracycline and ampicillin. Bile acid resistance was observed until 80 g/L Oxgall, corresponding to 80% bile (Table 1).

Based on the fact that strains DSM33407 and DSM 22607 share 99% of their 16S rRNA sequence and displayed similar microbiological characteristics, we concluded that strain DSM33407 is a new strain of the species *Christensenella minuta,* family *Christensenellaceae,* order Clostridiales, class Clostridia, phylum Firmicutes.

### 3.2. DSM33407 Protects from Diet-Induced Obesity and Regulates Associated Metabolic Markers

**Figure 2:**
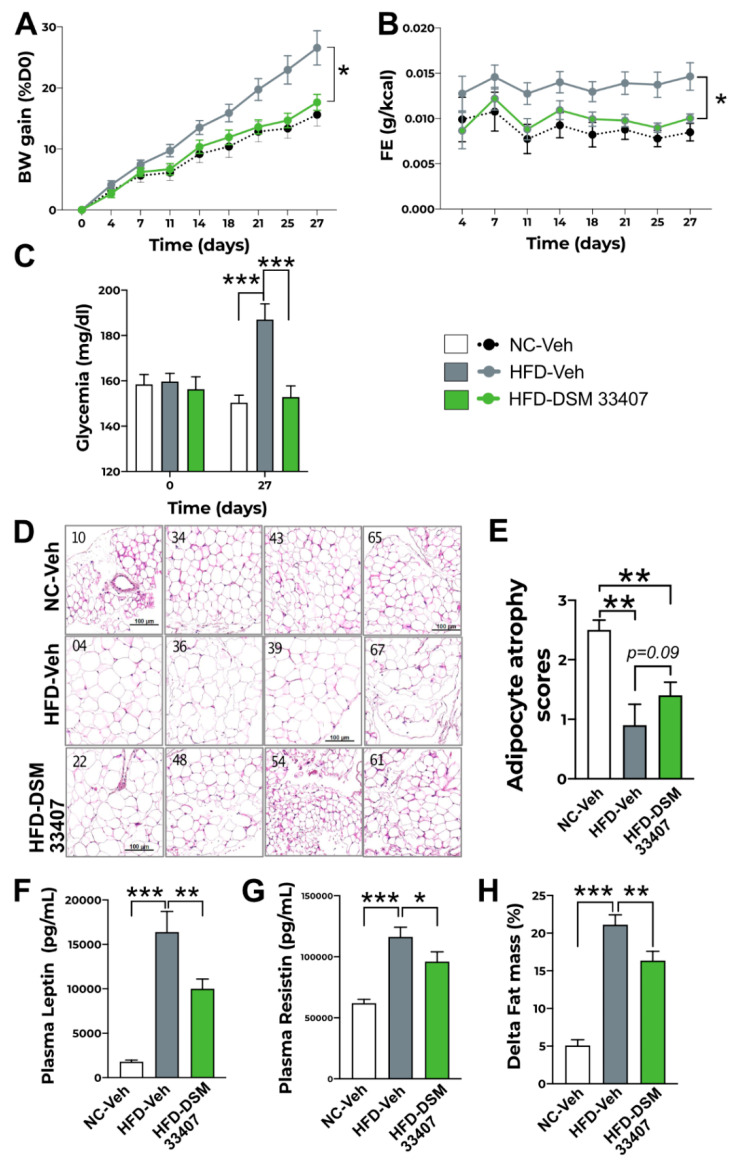
*C. minuta* DSM33407 prevents accumulation of visceral fat mass and hyperglycemia in a DIO mouse model (n = 10). (**A**) Growth curve of animals fed a normal chow (NC) or high-fat diet (HFD) and treated by oral gavage with a solution of vehicle (Veh) or *C. minuta* DSM33407 (DSM33407) for 4 weeks. (**B**) Associated feed efficiency over the 4-week treatment period. (**C**) Fasted glycemia was measured at baseline and at the end of the 4-week treatment period. (**D**) Representative pictures of mesenteric white adipose tissue hematoxylin and eosin staining after 40 days of treatment (scale bar: 100 mm) and associated adipocyte atrophy scores (**E**). Plasma leptin (**F**), resistin (**G**), and changes in fat mass (**H**) were measured after 12 weeks of treatment. *Key*: BW, Body Weight; FE, Feed Efficiency. *Statistics*: Two-way repeated measures ANOVA followed by two-stage linear step-up procedure of Benjamini, Krieger, and Yekutieli multiple comparisons (**A**–**C**), Mann–Whitney U test (**E**), one-way ANOVA followed by Tukey’s multiple comparisons (**F**,**G**); * *p* < 0.05, ** *p* < 0.01, *** *p* < 0.001.

Strain *C. minuta* DSM22607 has been shown to limit body weight gain and adiposity in a foundational work published in 2014 [2]. Thus, we evaluated the antiobesity potential of *C. minuta* DSM33407 in a diet-induced obesity (DIO) mouse model, where bacteria were administered daily (2.10^9^ CFU) by oral gavage for 4 to 12 weeks over four independent experiments, which returned similar results on body weight gain and metabolic parameters. Yet data were not pooled together, and only representative results are displayed in Figure 2. For other growth curves, refer to Supplemental Material (Supplementary Figure S2). HFD-induced body weight gain was prevented by DSM33407 and statistically indissociable from the normal diet control (Figure 2A). No differences were observed on daily food intake (Supplementary Figure S2). Feed efficiency (FE) (BW gain in g/total ingested calories in Kcal) was calculated to reflect the food metabolization rate and was observed to be significantly reduced during *C. minuta* DSM33407 administration (Figure 2B). This suggests that *C. minuta* DSM33407 does not influence feeding behavior but instead impacts food metabolism. Besides prevention of body weight gain, *C. minuta* DSM33407 also prevented diet-induced hyperglycemia (Figure 2C). At the macroscopic level, high-fat diet-induced hypertrophy of the mesenteric white adipose tissue (WAT) was significantly reduced when animals received *C. minuta* DSM33407 for 40 days (Figure 2D,E). Circulating leptin levels were significantly reduced after 12 weeks of *C. minuta* DSM33407 treatment (Figure 2F). This was also observed at 45 days (Supplementary Figure S2C). In addition, plasma resistin levels, another adipokine associated with chronic inflammation, were significantly reduced in *C. minuta* DSM33407-treated animals, indicating that the bacteria promoted a healthier WAT activity (Figure 2G). Finally, body composition measured after 12 weeks of treatment revealed a significant limitation of fat mass accumulation (Figure 2H) while lean mass was not significantly affected (Supplementary Figure S4A). Hence, these results indicate that *C. minuta* DSM33407 prevents the development of obesity in a DIO mouse model.

### 3.3. DSM33407 Modulates the Gut Microbiota and Their Metabolic Activity

**Figure 3:**
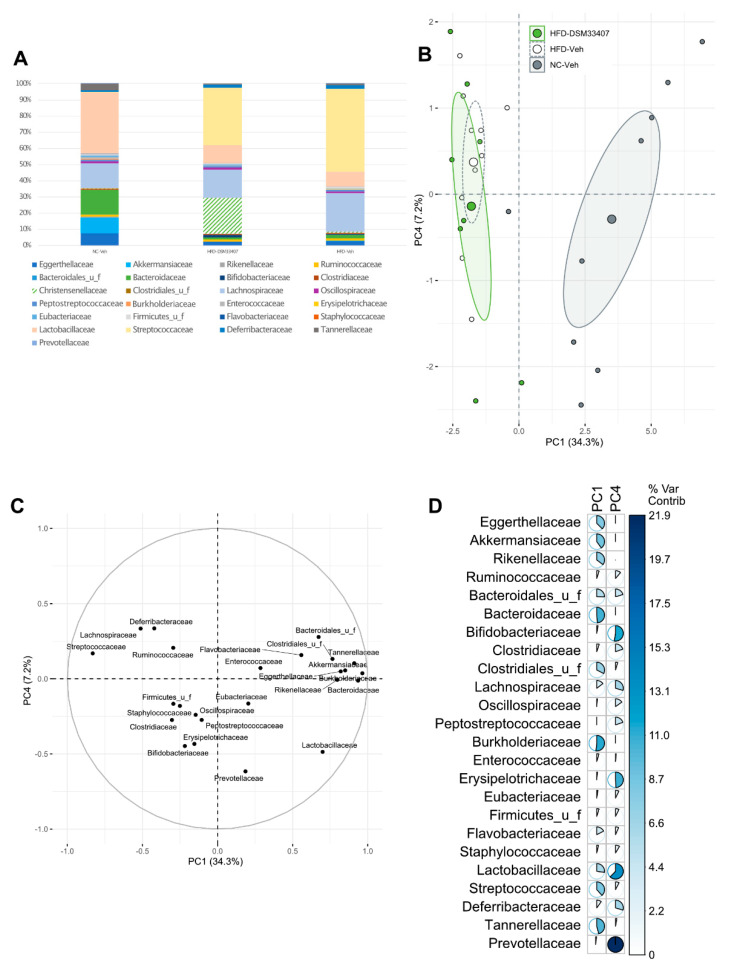
*C. minuta* DSM33407 modulated the fecal microbiome in a DIO mouse model assessed after 40 days of oral treatment (min n = 8). (**A**) Microbiome profile assessed using shotgun metagenomic profiling and displayed in a normalized stacked bar plot at the family level. (**B**) PCA analysis scores calculated from the dataset after removal of the *Christensenellaceae* family. The larger dot represents the group mean point surrounded by the 95% confidence ellipse. (**C**) PCA loadings plot and (**D**) associated variable contributions. The colored bar is scaled to the maximum percentage of contribution.

In order to elucidate the mechanisms of action underlying the antiobesity effects of *C. minuta* DSM33407, we first investigated its impact on the gut microbiota in the DIO mouse model used in the aforementioned preclinical studies using shotgun metagenomics in fresh feces after 40 days of treatment. The normalized bacterial community composition analyzed at the family level revealed that HFD drastically reduced the *Bacteroidaceae*, while the *Streptococcaceae* bloomed (Figure 3A). In the *C. minuta* DSM33407-treated group, the *Christensenellaceae* represented approximately 20% of the fecal microbiota (Figure 3A). As a consequence, the diversity of the gut microbiome, reflected by the Shannon index, was significantly reduced in this group (Supplementary Figure S4). In order to detect unbiased changes in the gut microbiome ecosystem induced by the presence of *C. minuta* DSM33407, we removed the *Christensenellaceae* from the dataset, renormalized it, and performed a principal component analysis (PCA) on the new matrix. Unsurprisingly, the largest source of variation (34.3%) was driven by diet (Figure 3B). A PERMANOVA analysis using Bray–Curtis distances indicated that the three groups were distinctly clustered (*p* = 0.047). A pairwise PERMANOVA adjusted with Bonferroni’s method revealed that only the HFD-Veh versus NC-Veh groups were significantly different (*p* = 0.003). Thus, the HFD-DSM33407 group was neither statistically distinguishable from the HFD-Veh, nor was it from the NC-Veh group, indicating that it is an intermediate state between the two control groups. This effect was captured on the fourth principal component (PC4) (7.2% of variance), where the fecal microbiome composition of the HFD-DSM33407 group co-located with the NC-Veh group. In particular, these two groups were characterized by higher levels of *Prevotellaceae, Lactobacillaceae, Erysipelotrichaceae*, and *Bifidobacteriaceae* compared with the HFD-Veh group (Figure 3B–D). On the contrary, the main bacterial families associated with the HFD-Veh group were the *Deferribacteraceae* and the *Lachnospiraceae* (Figure 3B–D). Therefore, this analysis shows that the DSM33407 treatment limited the HFD-induced microbial shift.

**Figure 4:**
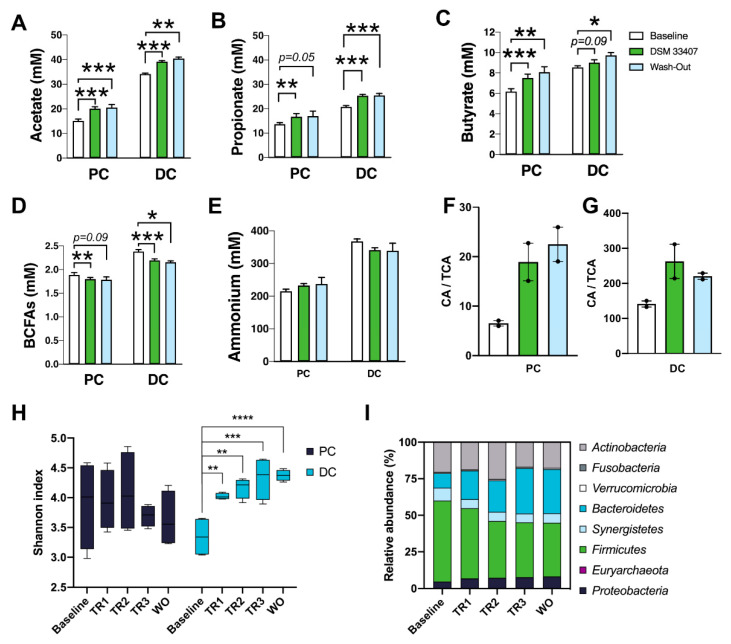
*C. minuta* DSM33407 improves gut microbiota diversity and changes microbial metabolism in a Simulator of the Human Intestinal Microbial Ecosystem (SHIME^®^) primed with human obese colonic microbiota. The main short-chain fatty acids acetate (**A**), propionate (**B**), and butyrate (**C**), as well as branched-chain fatty acids (BCFAs) (**D**) and ammonium (**E**) concentrations were measured in the proximal and distal colon (PC and DC, respectively) at three time-points, namely baseline (Baseline), after three weeks of treatment (DSM33407), and during the wash-out period (Wash out). Similarly, the ratio of unconjugated over conjugated forms of the primary bile acid cholic acid was measured in proximal (**F**) and distal (**G**) colon (n = 2). Shotgun metagenomics data were collected weekly at baseline, during the three weeks of treatment (TR1, TR2, and TR3, corresponding to first, second, and third week of treatment, respectively) and during wash-out (WO) to evaluate gut microbiota diversity using the Shannon Index (**H**) and relative abundance at the phylum level (**I**). Key: CA, Cholic Acid; TCA, Taurocholic Acid. *Statistics:* mixed REML followed by uncorrected Fischer’s LSD (**A**–**E**); H: Statistics done independently for each compartment. One-way ANOVA followed by Dunnett’s test with Baseline as control group. * *p* < 0.05, ** *p* < 0.01, *** *p* < 0.001, **** *p*< 0.0001.

In order to validate these findings in an independent and human-relevant model, we investigated the chronic interactions between *C. minuta* DSM33407 and the human gut microbiome in a SHIME^®^ model inoculated with fresh human feces [24]. Three donors with obesity were originally selected based on low levels of *Christensenella* sp. (data not shown). All three experiments were run in parallel and received *C. minuta* DSM33407 (2.10^9^ CFU/day). Weekly samples were collected to monitor production of key metabolites and to assess modulations of the microbiome community using shotgun metagenomic sequencing. Upon analysis of microbiota profiles, one donor (donor A) appeared to be largely dominated by *Akkermansia muciniphila* (Supplementary Figure S6A–C). In order to avoid any bias, this donor was discarded for the rest of the analysis. The data presented in Figure 4 are therefore the results of two experiments with independent donors with obesity. *C. minuta* was only detected during the treatment period, indicating that the bacteria were not able to engraft in the luminal compartment of the in vitro system (Supplementary Figure S6D,E). *C. minuta* DSM33407 was also mostly detected in the vessel corresponding to the proximal colon (Supplementary Figure S6D,E). Yet, a significant increase in short chain fatty acids (SCFAs; acetate, butyrate, and propionate) was detected in both compartments and this increase was sustained during the wash-out period (Figure 4A–C). In parallel, production of branched-chain fatty acids (BCFAs; isobutyric acid, isovaleric acid, and isocaproic acid), which result from the metabolism of branched-chain amino acids (BCAAs) and act as markers of bacterial proteolysis, was decreased (Figure 4D), while ammonium levels remained unchanged (Figure 4E). Bile acid changes were also monitored and an increase in the ratio of unconjugated over conjugated forms of the primary bile acid cholic acid (CA/TCA) upon *C. minuta* DSM33407 treatment was observed in both the proximal and distal colonic compartments (Figure 4F,G). Interestingly, the secondary bile acid litocholic acid (LCA) increased in the distal colon during the wash-out period (Supplementary Figure S6F,G). Together these observations indicate that *C. minuta* DSM33407 may promote the deconjugation of primary bile acids, since unconjugated bile acids are the substrate for secondary bile acid synthesis, such as LCA.

Concomitant to these metabolic changes, we observed a significant increase in the Shannon index of microbial community diversity in the distal colon as early as in the first week of treatment with *C. minuta* DSM33407, which was sustained during the wash-out period (Figure 4H). This increase in diversity was accompanied by a marked improvement in Bacteroidetes levels while Firmicutes levels declined (Figure 4I). Hence, both animal and in vitro human gut models indicated that *C. minuta* DSM33407 treatment had a profound influence on the gut bacterial community.

### 3.4. DSM33407 Associates with Modulations of Hepatic Lipid Metabolism

**Figure 5:**
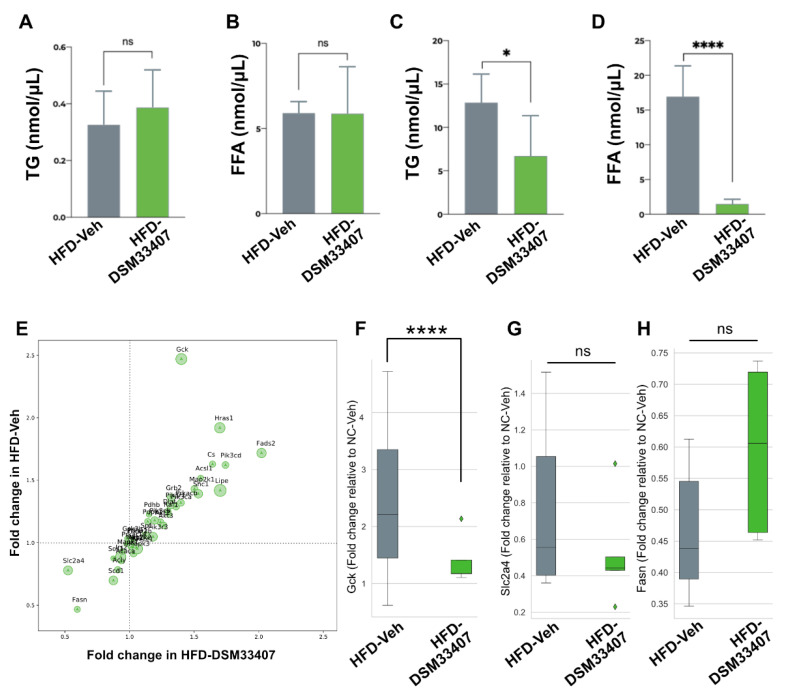
*C. minuta* DSM33407 modulates lipid metabolism in a diet-induced obesity mouse model. Fecal triglyceride (**A**) and free fatty acid (**B**) levels after 40 days of treatment (n = 10). Hepatic triglyceride (**C**) and free fatty acid (**D**) levels after 40 days of treatment (n = 10). (**E**) Hepatic gene expression analysis relative to NC-Veh control after 50 days of treatment (n = 5). Fold change values of Gck (**F**), Slc2a4 (**G**), and Fasn (**H**). *Key:* TG, Triglycerides; FFA, Free fatty acids. *Statistics:* (**A**–**C**) Mann–Whitney U test, (**D**) unpaired *t*-test; (**F**, **G**, and **H**) one-way ANOVA followed by two-stage linear step-up procedure of Benjamini, Kriger, and Yekutieli. * *p* < 0.05, **** *p* < 0.0001.

In order to gain some insights into the metabolic adaptations that were associated with *C. minuta* DSM33407 treatment during exposure to HFD, we first questioned whether the bacteria prevented intestinal lipid absorption. Fecal triglycerides and free fatty acids (FFA) were measured after 53 days of treatment and revealed no difference between the HFD-Veh and the HFD-DSM33407 groups (Figure 5A,B). We then evaluated the same markers in liver biopsies and observed a significant decrease of both hepatic triglycerides and FFA (Figure 5C,D). This indicated some specific regulation of hepatic lipid metabolism in the HFD-DSM33407 group that we further explored by assessing expression levels of a selected panel of hepatic genes involved in key metabolic pathways (Supplementary Table S1). As displayed on Figure 5E,F, the *Gck* gene coding for glucokinase was significantly repressed. Other genes such as *Slc2a4*, coding for the glucose transporter GLUT4, and *Fasn*, coding for the fatty acid synthase, tended to be regulated but these changes were not statistically significant (Figure 5G,H). This observation was independently replicated in a study where animals had been treated for 40 days with the same oral daily dose of *C. minuta* DSM33407 (Supplementary Figure S2). GCK is a key enzyme involved in the regulation of hepatic glycolysis that has also been linked with the regulation of lipogenesis [25]. Here, the strong decrease in hepatic FFA along *Gck* repression indicates that *C. minuta* DSM33407 may be involved in the regulation of hepatic lipid synthesis.

### 3.5. DSM33407 Maintains Gut Epithelial Integrity

**Figure 6:**
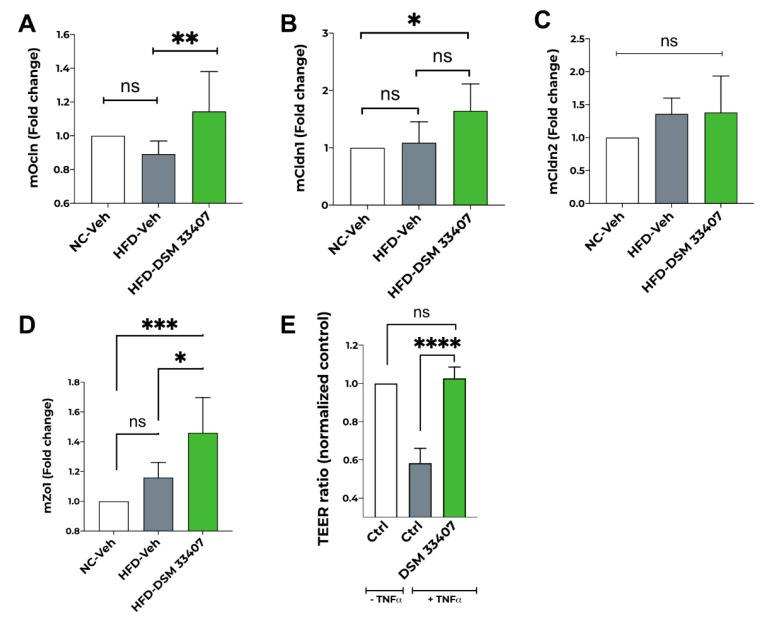
*C. minuta* DSM33407 maintains gut barrier integrity in both in vivo and in vitro preclinical models. Fold change expression of colonic tight junction proteins Occludin (*Ocln*) (**A**), Claudin 1 (*Cldn1*) (**B**), Claudin 2 (*Cldn2*) (**C**), and Zonula occludens-1 (*Zo1*) (**D**) in a DIO mouse model after 40 days of treatment (n = 6). (**E**) In vitro Trans-Epithelial Electrical Resistance (TEER) upon exposure to the proinflammatory cytokine TNF-a. *Statistics:* one-way ANOVA followed by Tukey’s (**A**–**D**) or Dunett’s (**E**) multiple comparisons; for all: * *p* < 0.05, ** *p* < 0.01, *** *p* < 0.001, **** *p* < 0.0001.

Finally, since several studies have shown that the DIO mouse model displays an altered gut epithelial membrane with increased permeability [26,27], we hypothesized that *C. minuta* DSM33407 may contribute to improve metabolic markers associated with obesity through restoration of the gut barrier. We first analyzed by RT-QPCR the expression of key tight junction proteins in the colon of DIO mice after 40 days of exposure to HFD and observed a significant increase in the expression of *Ocln* and *Zo1* in the HFD-DSM33407 group compared with untreated animals (Figure 6A,D). We also detected an increasing trend for *Cldn1* expression following DSM33407 treatment (Figure 6B), whereas expression of *Cldn2* remained unchanged (Figure 6C). We then investigated this further in vitro using a conventional model of barrier integrity based on transepithelial electrical resistance (TEER) and observed a strong protective effect of *C. minuta* DSM33407 (Figure 6E). Therefore, we concluded that *C. minuta* DSM33407 has good potential to act as a membrane barrier protector during high fat challenge.

## 4. Discussion

In this study, we described a new strain of the human commensal species *C. minuta* that was isolated from a healthy human donor. Using a standard API test system, the new strain *C. minuta* DSM33407 showed similar enzyme activities as those described for the original type strain *C. minuta* DSM22607, except for mannose, which has been reported as being weakly produced by the type strain and that we did not observe. *C. minuta* are sub-dominant Clostridiales species naturally present in the human gut of healthy individuals [28]. Because of their low abundance ranging from circa 0.2 to 2% of the gut microbiome, they tend to be overlooked in epidemiological studies, although a recent article established their prevalence to be around 50% in a Chinese cohort [6]. Nevertheless, the association between *C. minuta* and health has been largely documented [28]. One of the most prominent associations in human studies is an anticorrelation with obesity and related metabolic markers such as circulating cholesterol [5,14]. In addition, an early study demonstrated a causal link between the presence of *C. minuta* in the gut microbiota and lower adiposity in animal models [2]. Hence, we explored the antiobesity potential of this new strain of *C. minuta* and confirmed its potent antiobesity activity in a DIO mouse model. Since we neither detected any effects on food intake nor on fecal fat loss, but detected a significant reduction of food efficiency, we hypothesized that *C. minuta* DSM33407 regulates energy expenditure metabolically. Consistently, we observed that *C. minuta* DSM33407 blunted hepatic lipid accumulation in HFD-fed animals, which was associated with a strong repression of the *Gck* gene coding for hepatic glucokinase. The role of GCK in the regulation of body weight has been previously reported and its overexpression facilitates body weight gain through downregulation of thermogenic proteins in the brown adipose tissue (BAT) [29]. Indeed, the uncoupling protein UCP1 has long been known to induce adaptive adrenergic nonshivering thermogenesis [30] and is regulated by hepatic GCK through sympathetic nerve activity [31]. Logically, we tested the hypothesis that *Gck* downregulation may be associated with an upregulation of *Ucp1* expression and increased thermogenesis, but did not detect any differences in *Ucp1* expression in BAT (data not shown). Since other important actors of nonshivering thermogenesis have not been assessed yet in relation with *C. minuta* action, we believe that further analyses must be performed to make definitive conclusions about the potential involvement of *Gck*-mediated thermoregulation in relation with *C. minuta* DSM33407 treatment. In addition, the fact that only *Gck* was significantly repressed in these experiments indicates that it may be an early marker of hepatic response to *C. minuta* DSM33407 in this model. A comprehensive analysis of hepatic gene expression after a longer treatment period will be necessary to fully understand the impact of these bacteria on energy metabolism.

Consistent with reduced body weight gain, treatment with *C. minuta* DSM33407 was associated with lower fat mass and reduced hypertrophy of mesenteric WAT. This impact on fat mass was also reflected at the hormonal level through lower circulating levels of the adipokines leptin and resistin. Resistin is a polypeptide hormone mainly expressed and secreted by adipose tissue in mice, which plays an important role as a mediator in obesity-induced insulin resistance [32]. As previously reported [32], we observed a strong increase in circulating resistin levels in DIO mice, which was significantly hampered by *C. minuta* DSM33407 treatment. Since resistin was also shown to regulate glycemia, it is possible that the *C. minuta* DSM33407-related normalization of fasted glycemia in the DIO model may be partly mediated by the effect on resistin.

At the intestinal level, *C. minuta* DSM33407 minimized HFD-induced microbial shift. This observation was complemented in vitro, where we observed that chronic daily administration of DSM33407 significantly improved microbial diversity in the distal colon and restored the Firmicutes/Bacteroidetes ratio in a humanized SHIME^®^ model inoculated with obese fecal microbiota (Figure 4). Alteration of the Firmicutes/Bacteroidetes ratio is a hallmark of gut microbiota dysbiosis in obesity [33]. Restoring this ratio is a promising signal that the obesity-associated dysbiosis can be corrected using a single bacterial species. Nonetheless, restoring the metabolic functions of the dysbiotic microbiome must be the ultimate endpoint targeted by microbiome-based therapies. Therefore, we measured the major metabolic endpoints of the gut microbiome in the SHIME^®^ model. The *C. minuta*-induced microbial shift was associated with increased SCFAs and lower BCFAs levels in both the proximal and distal colon, indicating that DSM33407 stimulated carbohydrate fermentation and reduced proteolysis. Colonic SCFAs have been largely documented for their beneficial effects, such as the regulation of trophic functions at the gut epithelial barrier level that contribute to maintain a healthy intestinal membrane [34]. Interestingly, we evaluated *C. minuta* DSM33407 SCFA production and confirmed that the strain is a high acetic acid producer and moderate butyric acid producer (Supplementary Figure S7), as described for the type strain [1]. We did not detect any production of propionate, which corroborates that the increased SCFA levels are driven by a modulation of the metabolic activity of the microbial ecosystem following exposure to *C. minuta* DSM33407. Of interest, SCFAs are known to regulate systemic energy expenditure [35] and it is possible that they conveyed, at least in part, the strong effect we observed on hepatic lipid metabolism.

Finally, we observed that *C. minuta* DSM33407 improved the expression of intestinal tight junction proteins in vitro and in vivo. Increased intestinal permeability due to a loosening of tight junction proteins in obesity has been proposed as one of the driving factors of obesity-associated chronic low-grade inflammation [36,37]. Even if SCFAs, and particularly butyric acid, have been shown to restore intestinal membrane integrity [38], further analyses must be performed to evaluate whether SCFA production plays a role in the *C. minuta* DSM33407-induced tightening effect. Yet this is another encouraging observation pointing at a beneficial effect of *C. minuta* DSM33407 to restore intestinal health.

## 5. Conclusions

This work demonstrates that *C. minuta* DSM33407 limits body weight gain in a DIO mouse model and normalizes several metabolic markers upregulated as a consequence of obesity. We also addressed the mode of action of *C. minuta* DSM33407 and suggest it acts as a keystone bacterium, a subdominant species carrying unique functions to restore gut microbiota diversity in individuals with obesity, maintain gut epithelium integrity, and limit adiposity.

Altogether, these findings strongly support the therapeutic potential of *C. minuta* DSM33407 and warrant further clinical assessment. Hence, we are currently assessing the safety and tolerability of *C. minuta* DSM33407 in a Phase 1 clinical trial under FDA regulation.

## 6. Patents

Part of this work contributes to the data package for a pending patent (FR2009377). Dr. Mazier, Dr. Le Corf, Dr. Martinez, Dr. Soto, Dr. Elustondo, Dr. Rawadi, and Dr. Claus are part of the inventors list.

## Figures and Tables

**Table 1:** Physiological and biochemical properties of *C. minuta* DSM 33407.

**Strain Characteristics**	
Morphology	Short Rods with Tapered ends; Single, Pairs, or Rosettes
Growth condition	Anaerobic
Gram stain	Negative
Motility	None
Spore formation	None
Catalase	Negative
Oxidase	Negative
pH range	6.0 to 9.0
Bile resistance	Up to 80%
**API 20A Gallery Results**	
**Subtract**	**Growth**
Indole (IND)	−
Urea (URE)	−
Glucose (GLU)	+
Mannitol (MAN)	−
Lactose (LAC)	−
Saccharose (SAC)	−
Maltose (MAL)	−
Salicin (SAL)	+
Xylose (XYL)	+
Arabinose (ARA)	+
Gelatin (GEL)	−
Esculin (ESC)	−
Glycerol (GLY)	−
Cellobiose (CEL)	−
Mannose (MNE)	−
Melezitose (MLZ)	−
Raffinose (RAF)	−
Sorbitol (SOR)	−
Rhamnose (RHA)	+/−
Trehalose (TRE)	−

**Table 2:** Antibiotic resistance profile of *C. minuta* DSM 33407. *MIC*: Minimum Inhibitory Concentration.

Antibiotic Name	MIC (μg/mL)	Conclusion
Ampicillin	4	Resistant
Tetracycline	>32	Resistant
Chloramphenicol	8	Sensitive
Clindamycin	0.03	Sensitive
Meropenem	0.25	Sensitive
Metronidazole	0.12	Sensitive
Moxifloxacin	0.25	Sensitive
Piperacillin/Tazobactam	1/4	Sensitive

## Data Availability

Restrictions apply to the availability of these data. Data were obtained from Ysopia Bioscience and are available from the corresponding author with the permission of Ysopia Bioscience.

## Supplementary Materials

The following are available online at https://www.mdpi.com/article/10.3390/cells10040823/s1, Figure S1: Gram staining of new strain DSM 33407, Figure S2: Complementary data for the preclinical study performed in a DIO mouse model for 45 days (growth curve, feed efficiency, plasma leptin, and glycemia), Figure S3: Food intake measured after 27 days of daily oral treatment with *C. minuta* DSM 33407, Figure S4: Microbiome diversity observed after 53 days of treatment in a DIO mouse model, Figure S5: Complementary data for the preclinical study performed in a DIO mouse model for 12 weeks (lean mass, plasma adiponectin, inguinal adipocyte atrophy score, and glycemia), Figure S6: Complementary data for the SHIME^®^ experiment, Figure S7: Short-chain fatty acids produced by *C. minuta* DSM 33407 measured in the culture medium during stationary phase. Table S1: Hepatic gene expression in a DIO mouse model after 40 days of treatment.
