# Supplementary material for: A New Strain of Christensenella minuta as a Potential Biotherapy for Obesity and Associated Metabolic Diseases
Source: Cells. 2021 Apr 6;10(4):823. doi: 10.3390/cells10040823 (PMC8067450; doi:10.3390/cells10040823)
Supplement: Supplementary file 1 [file cells-10-00823-s001.pdf]

*Supplementary Materials*

# A New Strain of *Christensenella minuta* as a Potential Biotherapy for Obesity and Associated Metabolic Diseases

**Wilfrid Mazier [1], Katy Le Corf [1], Ccori Martinez [1], Héloïse Tudela [1], Déborah Kissi [1], Camille Kropp [1,2], Chrislain Coubard [1], Marion Soto [1], Frédéric Elustondo [1], Georges Rawadi [1] and Sandrine P. Claus [1,*]**

## Supplementary Methods

*Microbial characterization*

16S genotyping and phylogenetic analysis

The primers used for the 16S rRNA PCR amplification are universal primers (forward: 5′-AGA GTT TGA TCC TGG CTC AG-3′; reverse: 5′-ACG GCT ACC TTG TTA CGA CTT-3′).

Two out of the three 16S rRNA sequences identified in Morotomi & *al*. [3] as closest relative to *C. minuta* DSM 22607 based on 16S rRNA sequences were found in the top 22 hits of the blast analysis: *Caldicoprobacter oshimai* JW/HY-331 and *Tindallia californiensis* DSM 14871. The third one, which is the 16S rRNA sequence of *Clostridium ganghwense* JCM 13193, was downloaded from NCBI and added to the dataset.

This dataset of 25 16S rRNA sequences were aligned using Muscle from MEGA-X 10.1.8 [19]. The alignment was checked by 100 iterations using UPGMA (Unweighted Pair Group Method with Arithmetic mean). After the deletion of gaps and unknown bases, the Tamura-Nei model was used to construct the distance-matrix. The initial tree was obtained using neighbor joining and checked using the maximum likelihood method, using 1,000 iterations to validate the bootstrap values.

SCFA measurments

Bacterial supernatants were deproteinized over night at 4 °C with the addition of phosphotungstic acid (10%, Sigma). Then, they were centrifuged 15 min at 12000× *g*, and SCFA concentrations were determined by gas chromatography (Agilent 6890N Network) equipped with a split-splitless injector (GC Agilent 7890B), a flame-ionisation detector and a capillary column (15 m × 0,53 mm × 0,5 µm) impregnated with SP 1000 (Nukol; Supelco 25236). Carrier gas (hydrogen) flow rate was 10 mL/min and injector, column and detector temperatures were 200, 100 and 240 °C respectively. 2-Ethylbutyrate was used as the internal standard along standards of a panel of SCFAs. Samples were performed in duplicate and signals were integrated using the GC software (Agilent). To determine final SCFA concentration, supernatants were weighted before and after protein precipitation to obtain multiplier values corresponding to supernatant and sample weight ratio.

Transmission Electron Microscopy

10µL of bacterial solution were adsorbed on ionized carbon grids (Delta Microscopy, Toulouse, France) and negative staining was performed using Nano-Tungsten (Nano-W, Nanoprobes, LFG Distribution, France) (1 drop - 1min/drop). Grids were examined with a Transmission Electron Microscope (Talos F200S G2 - Thermofisher - Eindhoven) at 200kV, equipped with a 4K*4K camera One View (Gatan, Paris (France)).

## Sample collection and analysis from animal studies

*Animal housing and habituation*

120 animals were purchased from Charles River (France). Animals were singly housed (n=1/cage) in individually ventilated cages and allowed *ad libitum* access to water and food. Housing rooms were kept at constant temperature (22.0 ± 2.0 °C) and humidity (40–50 %) under a 12/12 light/dark cycle. The general appearance, behavior, welfare and

health of the animals were daily monitored throughout the study. At reception, all animals were fed the normal chow (NC, containing 8% of calories coming from fat; A04 diet, SAFE, France) for a two weeks acclimation period. The last week before initiating treatment, animals were habituated to the daily gavage procedure (saline 150 mL).

*Animal care and measurements*

In order to limit disruption in the microbiota caused by new clean litter and to avoid cage effect, a handful of mixed soiled litter (mixed from all individual cages from animals allocated to the same group) was always added to the new one.

Raw food intake (in grams) was measured by weighing the amount of food remaining in the food dispenser and calculating the difference with the previous measure. From this value, daily food intake was estimated by dividing the quantity by 3. Caloric intake (CI) was calculated using the diet energy content (NC= 3.34 kcal/g; HFD=4,7 kcal/g, Atwater factor). In addition, the feed efficiency (FE) was calculated as the ratio between the BW gain and the cumulative food intake (CI). Animal body composition was recorded at baseline (3 days before treatment initiation) and on the day of sacrifice, using a time domain nuclear magnetic resonance-based analyzer (LF90II, Bruker, Germany) in living and vigil animals.

*Sampling, Sacrifice and Tissue collection*

Blood samples were collected every week after a fasting period of 4 h using EDTA-K2 or heparin tubes, processed (3500rpm, 15min, 4 °C) and immediately stored at −80 °C. On the last day of experiment, animals received their last dose of treatment and were then fasted for 4 h before sacrifice. Then, a terminal blood sampling was collected by cardiac puncture on anaesthetized mice (Ketamine/Xylazine, 80/10 mg/kg). The whole liver and epidydimal white adipose tissue (WAT) were dissected out, weighed, snap-frozen into liquid nitrogen and stored at −80 °C. Blood glucose was measured from tail tip using glucometer (Glucofix Premium, Menarini Diagnostic).

Tissues used for histological analysis were isolated from the 6 weeks DIO study. Briefly, a terminal sampling consisted of neutral buffered formalin (NBF)-fixed and snap frozen mesenteric central and inguinal (subcutaneous) white adipose tissues (WAT). Tissues were processed to paraffin sections routinely stained with haematoxylin and eosin (HE). Microscopic changes and morphometric variables on adipocytes relative adipocyte atrophy scores were systematically monitored.

*Measures and evaluations*

Plasma levels of leptin, adiponectin and resistin were determined using custom BIO-RAD kits (Biorad, Marnes La Coquette France). All samples were analyzed in duplicate.

Fecal and liver Triglycerides (TG) and Free-Fatty Acids (FFA) quantification

Feces and liver samples (approximately 100 mg) were homogenized manually with a micropestle in 1 mL of ultrapure water with 5% NP-40 and incubated 5 min at 95°C. Samples were then put at room temperature to cool down et incubated a second time to allow the solubilization of the TG and FFA. Supernatants containing TG and FFA were separated from the residues by centrifugation. TG and FFA quantification were performed on the supernatants according to the manufacturer's protocols (Sigma-Aldrich MAK266 for TG and MAK044 for FFA). TG and FFA concentrations were determined by measurement of the absorbance at 570 nm (FLUOstar Omega, BMG Labtech).

*RNA extraction from liver, brown adipose tissue and proximal colon tissues*

RNA extraction was performed on approximately 50 mg of liver homogenized in 900 µL of Qiazol with one stainless steel bead (5 mm mean diameter per 2 mL microcentrifuge tube) using a Retsch MM400 (Retsch) for twice 2 min at 20 Hz. The gDNA Eliminator solution was added to eliminate genomic DNA contamination, making further treatment with DNase unnecessary and chloroform was added for phase separation. The upper

phase was purified on RNeasy Mini spin column and RNA was eluated with RNase-free water. All RNA samples were stored at −80 °C in nuclease-free microcentrifuge tubes until use.

*RNA quantification and quality assessment*

The RNA concentrations were determined by spectrophotometric measurement (NanoDrop One, ThermoFisher Scientific) and their quality evaluated using a RNA 6000 Nanochip with Agilent 2100 BioAnalyzer (Agilent), according to the manufacturer's instructions. Briefly, denatured RNA samples were loaded on RNA chip, which were primed with a mixture of gel-stain solution and loading buffer. The chip was run on 2100 BioAnalyzer station and the data analyzed by evaluation of the electropherogram tab with the 2100 Expert Software (Agilent).

*Reverse Transcription (RT) to generate cDNA and Real-Time Quantitative PCR (qPCR)*

100 ng of each total RNA preparation was used in an iScript Advanced cDNA synthesis reaction mix, primed with random and oligo(dT) primers for the Reverse Transcription. The RT reaction was performed following the manufacturer's instructions (Bio-Rad): 20 min at 46 °C for the RT and 1 min at 95 °C for RT inactivation. SsoAdvanced SYBR Green Universal Supermix (Bio-Rad) was used for qPCR reaction. All reactions were acquired on a CFX 96 Touch instrument (Bio-Rad) in duplicate in the pre-designed plate reference 10030779 loading with specific primers from Bio-Rad or in 96-multiwell plates (Bio-Rad). Each 20 µL reaction contained also 10 µL of SsoAdvanced SYBR Green Universal Supermix and 1 µL PrimePCR PreAmp Assay (Bio-Rad unique assay ID: *mGck* (qMmuCID0026296), *mGapdh* (qMmuCED0027497), *mHrpt* (qMmuCID0005679), *mTbp* (qMmuCID0040542) or *mCldn1*(qMmuCED0045581), *mCldn2* (qMmuCED0004515), *mOcln* (qMmuCED0046496) and *mZo1* (qMmuCID0005277)). The thermal cycling protocol started with polymerase activation at 95 °C for 2 min, followed by 40 cycles of denaturation at 95 °C for 5 s, annealing/extension at 60 °C for 30 s and ended with melt curve analysis for 5 s with 0.5 °C increment steps from 65 to 95 °C. Cq values were determined with the CFX Maestro software (Bio-Rad). All data were normalized to the expression of *Gapdh* and data analysis was performed with Python software.

In vitro *permeability assay*

Caco-2 cells were obtained from the ECACC through Sigma-Aldrich. The cells were grown in DMEM (Gibco) supplemented with 20% FBS at 37 °C in 5% $CO_2$ on 24-well Transwell insert filter (polyester membrane with 0.4 µm pore size, Costar, Corning Life Science) at a density of $3.10^4$ cells per well. The medium was changed every two days until the optimal transepithelial electrical resistance (TEER 2000 W/$m^2$) was reached using an EVOM3 ohmmeter (WPI). At this point, fresh DMEM medium was added and cells were treated in the apical compartment with bacteria at a ratio of 50:1, 3 h before adding 100 ng/mL of TNF-a (Invivogen) in the basal compartment. The TEER was measured before TNF addition (T0) and 6 h after (T6). Results were normalized to the basal condition.

**Triple SHIME® model**

The SHIME® (Simulator of Human intestinal Microbial Ecosystem; Prodigest, Belgium) is a dynamic in vitro model of the human gastrointestinal tract that offers to simulate the intestinal microbial processes that occur during digestion [21,38]. This model allows to culture complex gut microbiota over a long period under representative conditions of the different intestinal compartments.

Each SHIME® unit was inoculated in the proximal colon with one freshly collected human fecal microbiota coming from three preselected obese donors (BMI >30kg/$m^2$). Upon inoculation with the faecal microbiota, the colonic vessels were stabilized to simulate the proximal colon (PC; pH 5.6-5.9; retention time = 20h; volume of 500 mL) and distal colon (DC; pH 6.6-6.9; retention time = 32h; volume of 800 mL). The protocol started by a two weeks stabilization period allowing the microbial community to differentiate in the

different reactors depending on the local environmental conditions. Once systems stabilized, a control period of two weeks was initiated during which sampling of intestinal vessels was started in order to determine the baseline microbial community composition and activity, which was used as a reference to evaluate treatment effect (referred to in text as "Baseline"). Daily treatment with DSM 33407 was operated once a day for 3 weeks by adding $2.10^9$ CFU directly in the proximal colon vessel (liquid formulation, PBS 1×, 1% glycerol, total volume of 1ml per day). Concentrations of short chain fatty acids (SCFAs; acetate, propionate, butyrate), Branched Chain Fatty Acids (BCFAs; isobutyrate, isovalerate and isocaproate) and ammonium [39] were monitored during the assay. Bile acid analysis was performed by UHPLC-equipped QTrap 5500 mass spectrometer for LC-MS/MS analysis. Presence and putative engraftment of DSM 33407 was assessed by qPCR.

**Additional information about DNA library preparation for shotgun metagenomic sequencing**

The standard protocol was used for DNA input of 0.3–1.0 ng and a low input protocol was used for samples ≤0.2 ng of DNA. Genomic DNA was fragmented using a proportional amount of Illumina Nextera XT fragmentation enzyme. Dual indexes (i5 and i7) were added to each sample followed by 12 cycles of PCR for the standard protocol and 15 cycles of PCR for the low input protocol to construct libraries. DNA libraries were purified using AMpure magnetic Beads (Beckman Coulter) and eluted in QIAGEN EB buffer. DNA libraries were quantified using Qubit 4 fluorometer and Qubit™ dsDNA HS Assay Kit.

**Univariate statistical analysis**

Univariate statistics were performed with Prisms 8 (Graphpad). Normality distribution was assessed using Shapiro-Wilk or Kolmogorov-Smirnov test. Considering inter-individual variability in body weight gain in response to HFD, an outlier identification (Grubb's test) was systematically ran on normalized BW gain data. Animals with aberrant body weight changes were discarded from analysis. Data from body weight changes, food efficiency, and glycemia assessment were analyzed by two-way ANOVA repeated measures followed by Dunnett's multiple comparisons or Fischer's LSD test. Data from body composition and plasma markers were analyzed by One-way ANOVA followed by Holm-Sidak's multiple comparisons. Data from histological scoring were analyzed using pair-wise Mann Whitney tests. Regarding SHIME data, due to different number of samples depending on timepoints repeated measures ANOVA could not be handled. Therefore, analyses were made fitting a mixed model of Restricted Maximum Likelihood (REML), followed by uncorrected Fischer's LSD tests. For all analyses, the significance threshold was placed at $p = 0.05$. In the text and figures, data are represented as mean +/− SEM.

**Supplementary figures**

**S1:** Gram staining of new strain DSM 33407.

**S2:** Complementary data for the preclinical study performed in a DIO mouse model for 45 days (growth curve, feed efficiency, plasma leptin and glycemia).

**S3:** Food intake measured after 27 days of daily oral treatment with *C. minuta* DSM 33407.

**S4**: Microbiome diversity observed after 53 days of treatment in a DIO mouse model

**S5:** Complementary data for the preclinical study performed in a DIO mouse model for 12 weeks (lean mass, plasma adiponectin, inguinal adipocyte atrophy score and glycemia).

**S6:** Complementary data for the Triple-SHIME experiment

**S7:** Short Chain Fatty Acids produced by *C. minuta* DSM 33407 measured in the culture medium during stationary phase

TABLE S1: Hepatic gene expression in a DIO mouse model after 40 days of treatment

**Fasta sequence of the 16S rDNA fragment used for the phylogeny analysis presented in Figure 1.**

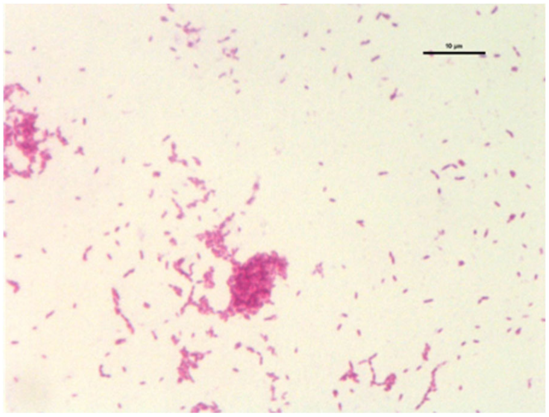

**Figure S1.** Gram staining of DSM 33407. Scale bar: 10 mm.

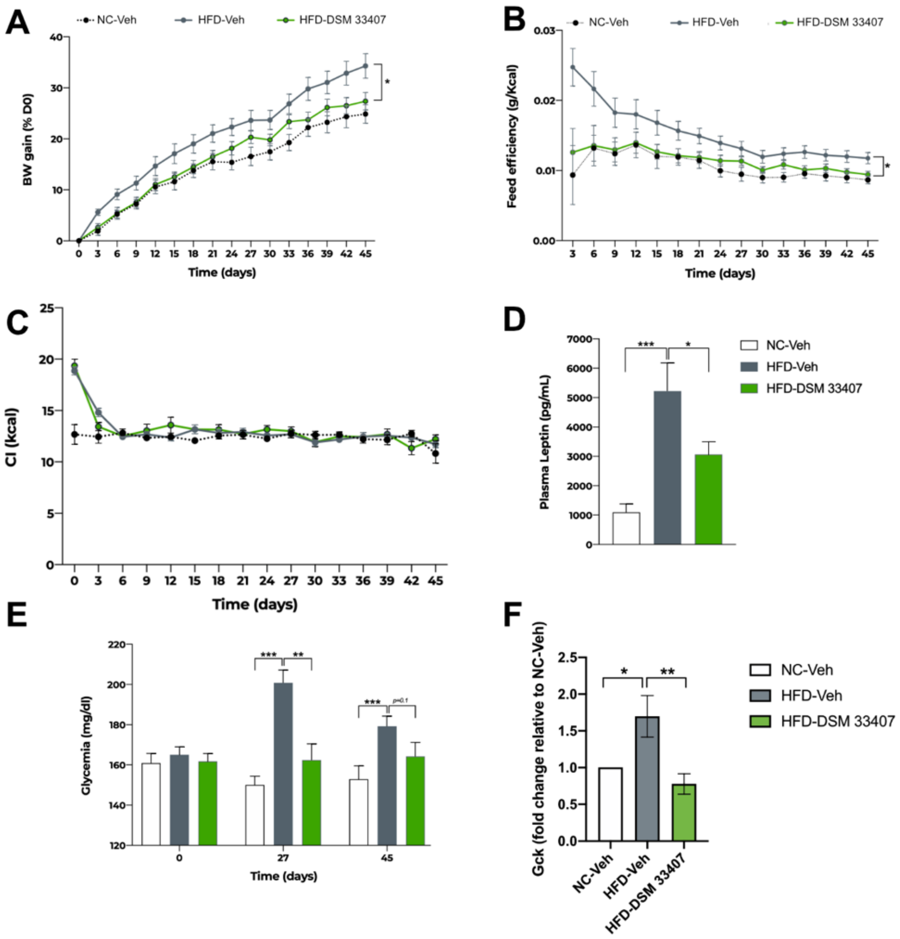


**Figure S2.** Complementary data for the preclinical study performed in a DIO mouse model for 45 days. **A.** Body weight (BW) gain of animals treated for 45 days with *C. minuta* DSM 33407 under normal chow (NC) or high fat diet (HFD) (n=10). **B.** Daily feed efficiency (FE) (n=10). **C.** Daily calorie intake (CI) (n=10). **D**. Plasma leptin (n=10). **E.** Fasted glycemia (n=10). **F**. Glucokinase (*Gck*) expression levels (n=5). Statistics: (A, B, C and E): Two-way repeated measures ANOVA followed by Two-stage linear step-up procedure of Benjamini, Krieger and Yekutieli multiple comparisons. (D

and F): One way ANOVA followed by Tukey's multiple comparisons. * p<0.05, ** p<0.01, *** p<0.001.

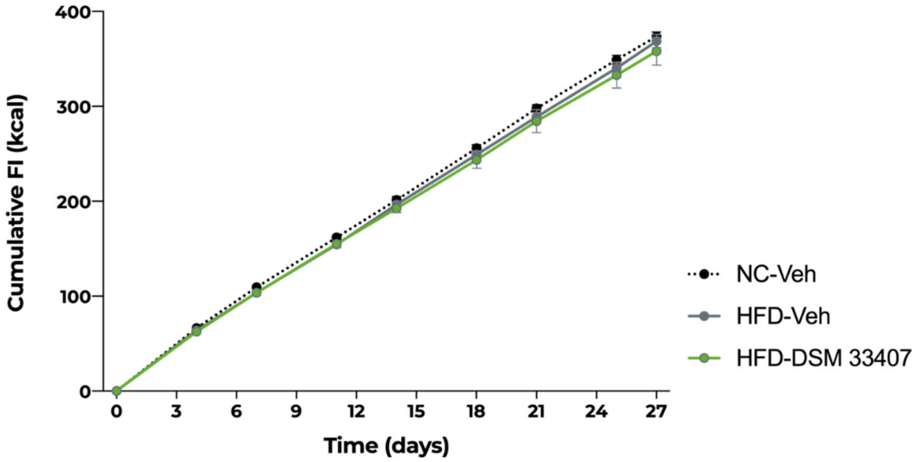


**Figure S3.** Food intake measured after 27 days of daily oral treatment with *C. minuta* DSM 33407. n=10. *Statistics*: Two-way repeated measures ANOVA followed by Two-stage linear step-up procedure of Benjamini, Krieger and Yekutieli multiple comparisons. * p<0.05, ** p<0.01, *** p<0.001.

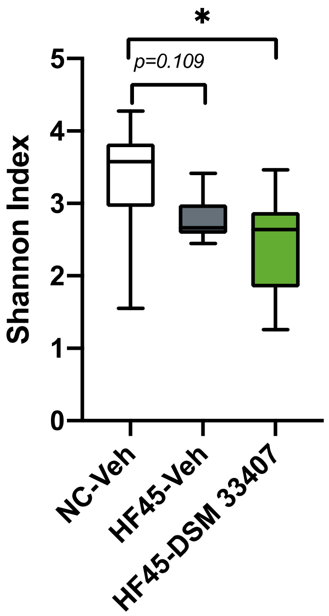


**Figure S4.** Microbiome diversity observed after 40 days of treatment in a DIO mouse model (min n=8). ANOVA (*p = 0.02*) followed by Dunnet's test with NC-Veh as fixed comparator group.

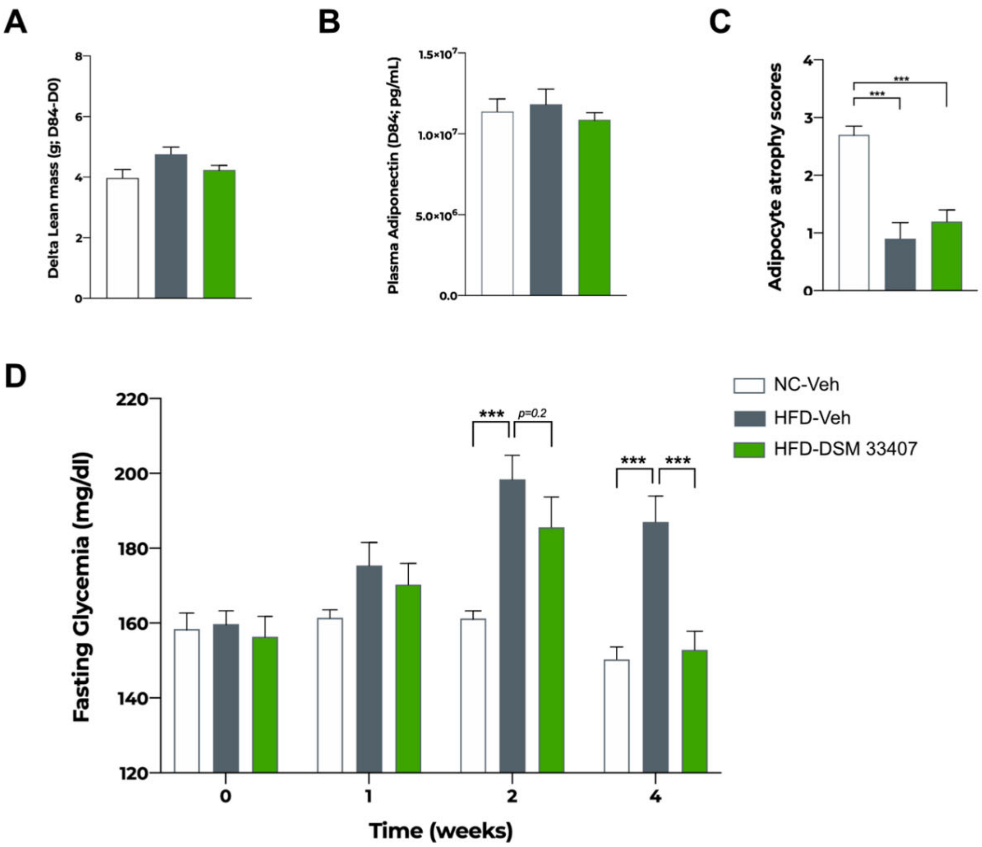


**Figure S5.** Complementary data for the preclinical study performed in a DIO mouse model for 12 weeks. Changes in lean mass (**A**). Plasma adiponectin (**B**). Inguinal adipocyte atrophy scores (**C**). Weekly fasted glycemia (**D**). *Statistics*: (A to C) One way ANOVA followed by Tukey's multiple comparison test; (D) Two-way repeated measures ANOVA followed by Two-stage linear step-up procedure of Benjamini, Krieger and Yekutieli multiple comparisons. * $p<0.05$, ** $p<0.01$, *** $p<0.001$.

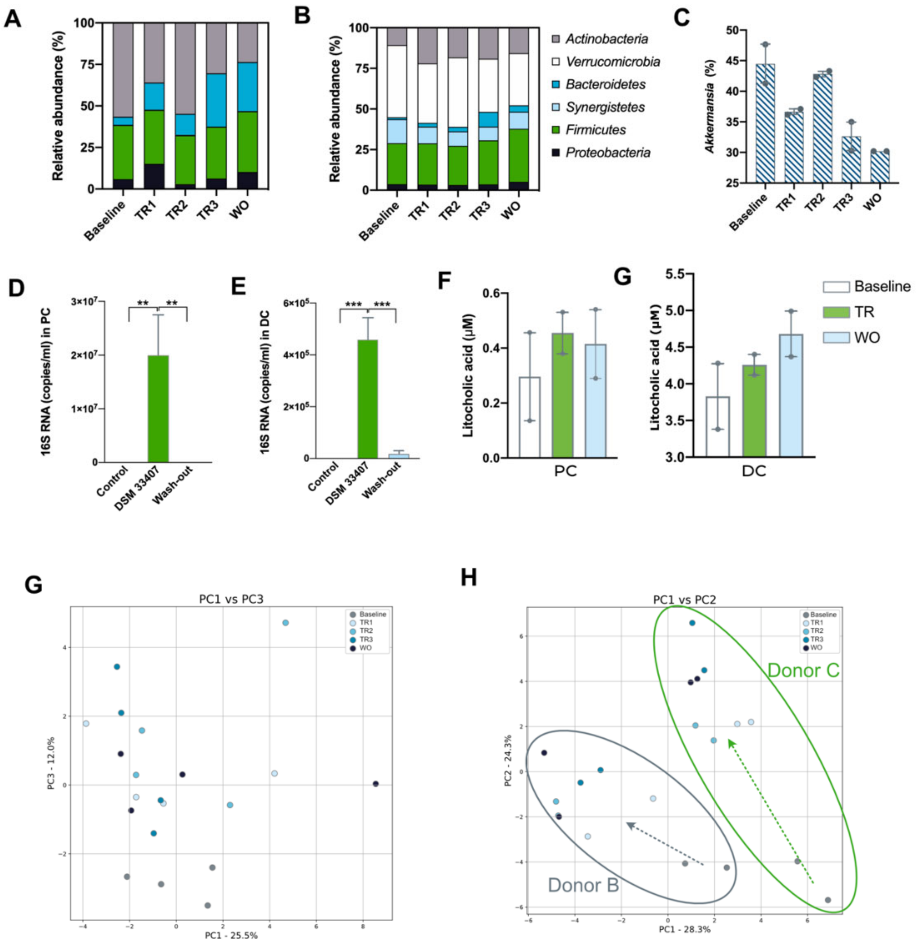


**Figure S6.** Fecal microbiota analysis from Triple-SHIME experiments. Microbiota phylum relative abundance for donor A in proximal colon (PC) (**A**) and distal colon (DC) (**B**). Levels of *Akkermansia* spp. in % of total detected genera in donor A (**C**). Detection of *Christensenella* spp. in PC (**C**) and DC (**D**) at each sampling time-point (n=6). Litocholic acid levels in PC (**E**) and DC (**F**) (n=2). Principal component analysis of microbiota composition at the family level in PC (**G**) and DC (**H**). *Statistics*: (D and E) One-way ANOVA followed followed by Tukey's multiple comparison test. * p<0.05, ** p<0.01, *** p<0.001.

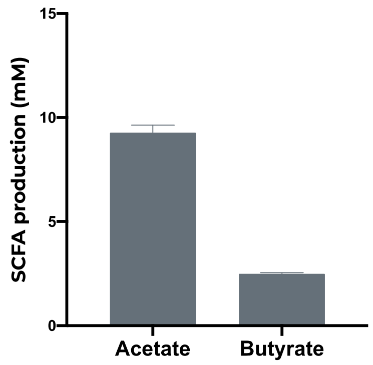


**Figure S7.** Short Chain Fatty Acids produced by *C. minuta* DSM 33407 measured in the culture medium during stationary phase. Propionate was not detected. (mean +/- sd from 2 experiments). SCFA: Short Chain Fatty Acids.

**Table S1.** Hepatic gene expression (fold change compared to NC-Veh group) in a DIO mouse model after 40 days of treatment. Statistics: Two-way ANOVA followed by two-stage linear step-up procedure of Benjamini, Kriger and Yekutieli (n=5).

| | HFD Veh | | HFD DSM 33407 | | | |
|---|---|---|---|---|---|---|
| **Gene** | **Mean** | **sd** | **Mean** | **Sd** | **Mean Diff.** | **adj. p value** |
| *Acaca* | 0.856 | 0.056 | 0.933 | 0.133 | -0.077 | 0.6793 |
| *Acly* | 0.784 | 0.048 | 0.909 | 0.145 | -0.125 | 0.5015 |
| *Acsl1* | 1.517 | 0.340 | 1.550 | 0.121 | -0.033 | 0.8597 |
| *Akt1* | 0.963 | 0.106 | 1.030 | 0.138 | -0.067 | 0.7205 |
| *Akt2* | 1.164 | 0.097 | 1.239 | 0.164 | -0.075 | 0.6859 |
| *Akt3* | 1.139 | 0.112 | 1.264 | 0.155 | -0.126 | 0.5003 |
| *Cs* | 1.628 | 0.169 | 1.642 | 0.141 | -0.014 | 0.9411 |
| *Dlat* | 1.252 | 0.094 | 1.298 | 0.131 | -0.046 | 0.8067 |
| *Fads2* | 1.718 | 0.295 | 2.021 | 0.311 | -0.303 | 0.1038 |
| *Fasn* | 0.466 | 0.110 | 0.596 | 0.136 | -0.129 | 0.4878 |
| *Gck* | 2.470 | 1.611 | 1.401 | 0.427 | 1.069 | <0,0001*** |
| *Grb2* | 1.361 | 0.229 | 1.321 | 0.196 | 0.039 | 0.8328 |
| *Gsk3b* | 1.034 | 0.098 | 0.985 | 0.112 | 0.049 | 0.7907 |
| *Hras1* | 1.919 | 0.650 | 1.698 | 0.387 | 0.221 | 0.2355 |
| *Irs1* | 0.960 | 0.096 | 1.052 | 0.127 | -0.092 | 0.6215 |
| *Irs2* | 0.886 | 0.472 | 0.931 | 0.369 | -0.045 | 0.809 |
| *Lipe* | 1.418 | 0.186 | 1.702 | 0.501 | -0.283 | 0.1286 |
| *Map2k1* | 1.429 | 0.257 | 1.502 | 0.174 | -0.073 | 0.696 |
| *Map2k2* | 0.966 | 0.174 | 1.013 | 0.144 | -0.047 | 0.8008 |
| *Mapk1* | 0.929 | 0.077 | 0.944 | 0.118 | -0.015 | 0.9349 |
| *Mapk3* | 0.915 | 0.102 | 1.031 | 0.186 | -0.116 | 0.5349 |
| *Pdha1* | 1.023 | 0.104 | 1.023 | 0.131 | 0.000 | 0.9987 |
| *Pdhb* | 1.226 | 0.120 | 1.151 | 0.108 | 0.075 | 0.6854 |
| *Pdpk1* | 1.169 | 0.058 | 1.141 | 0.138 | 0.029 | 0.8783 |
| *Pik3ca* | 1.289 | 0.113 | 1.360 | 0.169 | -0.070 | 0.7051 |
| *Pik3cb* | 1.179 | 0.122 | 1.195 | 0.199 | -0.016 | 0.9329 |
| *Pik3cd* | 1.621 | 0.328 | 1.742 | 0.166 | -0.121 | 0.5159 |
| *Pik3r1* | 1.303 | 0.526 | 1.318 | 0.232 | -0.015 | 0.9371 |
| *Pik3r3* | 1.048 | 0.056 | 1.182 | 0.279 | -0.134 | 0.4727 |
| *Prkacb* | 1.320 | 0.131 | 1.397 | 0.172 | -0.078 | 0.6769 |
| *Prkar1a* | 0.990 | 0.062 | 0.978 | 0.110 | 0.012 | 0.9495 |
| *Prkar2b* | 1.015 | 0.294 | 1.019 | 0.195 | -0.004 | 0.9833 |
| *Raf1* | 1.228 | 0.097 | 1.303 | 0.110 | -0.075 | 0.6872 |
| *Scd1* | 0.698 | 0.429 | 0.875 | 0.275 | -0.177 | 0.3421 |
| *Shc1* | 1.392 | 0.168 | 1.532 | 0.261 | -0.141 | 0.4499 |
| *Slc2a4* | 0.778 | 0.497 | 0.524 | 0.293 | 0.254 | 0.1727 |
| *Sos1* | 0.875 | 0.045 | 0.874 | 0.092 | 0.000 | 0.9988 |
| *Sp1* | 1.060 | 0.092 | 1.125 | 0.194 | -0.066 | 0.7246 |
| *Srebf1* | 0.951 | 0.148 | 1.061 | 0.375 | -0.109 | 0.5578 |

Key: *Acaca* Acetyl-CoA Carboxylase Alpha, *Acly* ATP Citrate lyase, *Acsl1* Acyl-CoA Synthetase Long Chain Family Member 1, *Akt1* AKT serine/threonine kinase 1, *Akt2* AKT serine/threonine kinase 2, *Akt3* AKT serine/threonine kinase 3, *Cs* Citrate synthase, *Dlat* Dihydrolipoamide Acetyl-transferase, *Fads2* Fatty acid desaturase 2, *Fasn* Fatty acid synthase, *Gck* Glucokinase, *Grb2* Growth factor receptor-bound protein 2, *Gsk3b* Glycogen synthase kinase 3 beta, *Hras1* Harvey murine sarcoma virus oncogene, *Irs1* Insulin receptor 1, *Irs2* Insulin receptor 2, *Lipe* Lipase E, *Map2k1* Mitogen-activated protein kinase kinase 1, *Map2k2* Mitogen-activated protein kinase kinase 2, *Mapk1* Mitogen-activated protein kinase 1, *Mapk3* Mitogen-activated protein kinase 3, *Pdha1* Pyruvate dehydrogenase E1 component subunit alpha, *Pdhb* Pyruvate dehydrogenase E1 component subunit beta, *Pdpk1* 3-phosphoinositide-dependent protein kinase 1, *Pik3ca* Phosphatidylinositol 4,5-bisphosphate 3-kinase catalytic subunit alpha isoform, *Pik3cb* Phosphatidylinositol 4,5-bisphosphate 3-kinase catalytic subunit beta isoform, *Pik3cd* Phosphatidylinositol 4,5-bisphosphate 3-kinase catalytic subunit delta isoform, *Pik3r1* Phosphatidylinositol 3-kinase regulatory subunit alpha, *Pik3r3* Phosphatidylinositol 3-kinase regulatory subunit gamma, *Prkacb* cAMP-dependent protein kinase catalytic subunit beta, *Prkar1a* cAMP-dependent protein kinase type I-alpha

regulatory subunit, *Prkar2b* cAMP-dependent protein kinase type II-beta regulatory subunit, *Raf1* leukemia viral oncogene homolog 1, *Scd1* stearoyl-CoA desaturase-1, *Shc1* Src homology 2 domain containing transforming protein 1, *Slc2a4* solute carrier family 2 member 4, *Sos1* son of sevenless homolog 1, *Sp1* Sp1 transcription factor, *Srebf1* sterol regulatory element binding transcription factor 1.

**Fasta sequence of the 16S rDNA fragment used for the phylogeny analysis presented in Figure 1.**

>RCB10F-16SF_P15

CCCTGCGCGTGCTTACCATGCAAGTCGAACGAGGTTGCCCTTT-
GTGAATCCTTCGGGAG-
GAACTGTGGGTATACCGAGTGGCGGACGGGTGAGTAACGCGTGAGCAACCTGC
CCTGCAACGGGGGACAACAGTTGGAAACGACTGCTAATACCGCATGAGACCAC-
GAAACCG-
CATGGTTTTGAGGTAAAAGGATTTATTCGATGCAGGATGGGCTCGCGTCCCATTA
GATAGTTGGTGAGGTAACGGCCCACCAAGTCAACGATGGGTAGCCGACCTGA-
GAGGGTGATCGGCCACACTGGAACTGAGACACGGTCCAGACTCCTACGGGAGG
CAGCAGTGGGGAATATTGGGCAATGGGGGAAACCCTGACCCAGCAAC-
GCCGCGTGAGGGAA-
GAAGGTCTTCGGATTGTAAACCTTTGTCCTATGGGACGAAAGAAATGACGGTAC
CATAGGAGGAAGCTCCGGCTAACTACGTGCCAGCAGCCGCGGTAATAC-
GTAGGGAGCAA-
GCGTTGTCCGGAATTACTGGGCGTAAAGGGTGCGTAGGTGGTCATGTAAGTCAG
ATGTGAAAGACCGGGGCTTAACCCCGGGGTTGCATTTGAAACTGTGTGACTT-
GAGTACAGGA-
GAGGGAAGTGGAATTCCTAGTGTAGCGGTGAAATGCGTAGATATTAGGAGGAA
CACCAGTGGCGAAGGCGACTTTCTGGACTGTAACTGACACTGAGGCACGAAA-
GCGTGGGGAG-
CAAACAGGATTAGATACCCTGGTAGTCCACGCCGTAAACGATGGATACTAGGTG
TGGGGCCCGATAGGGTTCCGTGCCGAAGCAAAC-
GCATTAAGTATCCCGCCTGGGGAGTAC-
GATCGCAAGGTTGAAACTCAAAGGAATTGACGGGGGCCCGCACAAGCAGCGGA
GCATGTGGTTTAATTCGAAGCAACGCGAAGAACCTTACCAAGGCTT-
GACATCCTCTGAC-
GACTGTAGAGATACAGTTTCNCTCCGCGCAAAGAGACAGGTGGTNCATGGCTGT
CGNTCAACTCGTGTCGTAAGATGTNGGTTAGTCCCGCAACAAGTCAACTCT-
NTGGTAGTAGCAACTNNTAAAGCCGGAATTCAGTGAGATGCCGGGGAA
